# Review of human papillomavirus (HPV) burden and HPV vaccination for gay, bisexual, and other men who have sex with men and transgender women in the United States

**DOI:** 10.1080/21645515.2021.2016007

**Published:** 2022-03-16

**Authors:** Elissa Meites, Timothy J. Wilkin, Lauri E. Markowitz

**Affiliations:** a Division of Viral Diseases, National Center for Immunization and Respiratory Diseases, Centers for Disease Control and Prevention, Atlanta, GA, USA; b Division of Infectious Diseases, Weill Cornell Medicine, New York, NY, USA

**Keywords:** Papillomavirus infections, papillomavirus vaccines, vaccination coverage, sexual and gender minorities

## Abstract

Gay, bisexual, and other men who have sex with men (MSM) and transgender women, particularly those who are living with HIV, are disproportionately affected by human papillomavirus (HPV). For this narrative review of HPV health outcomes and vaccination for gay, bisexual, and other MSM and transgender women in the United States, we highlighted 71 publications regarding 1) burden of HPV infections and related diseases; 2) HPV vaccine efficacy; 3) HPV vaccination recommendations; 4) HPV vaccination coverage; 5) real-world vaccine effectiveness and health impacts; and 6) HPV vaccination acceptability. Vaccination is effective at reducing HPV infections among MSM; in the United States, routine HPV vaccination is recommended for all adolescents at age 11–12 years and for all persons through age 26 years. Efforts are ongoing to increase vaccination coverage and monitor health impacts of vaccination. Increasing vaccination coverage before sexual exposure to HPV is expected to reduce the burden of HPV-related disease.

## Introduction

Gay, bisexual, and other men who have sex with men (MSM) and transgender women experience substantial health inequities,^1^including being disproportionately affected by human papillomavirus (HPV), the most common sexually transmitted infection in the United States.^2^ Although most HPV infections are asymptomatic, persistent infections with high-risk HPV types cause anogenital cancers, including cervical, vulvar, vaginal, anal, and some penile cancers, as well as oropharyngeal cancer; HPV also causes cancer precursor lesions such as anal intraepithelial neoplasias (AIN).^3^ An estimated 45,300 new cases of HPV-associated cancers occur annually in the United States, including about 25,400 among women, and about 19,900 among men.^4^ While the most common HPV-associated cancer among women is cervical cancer, the most common among men is oropharyngeal cancer.^4^ Other HPV infections can cause anogenital warts and recurrent respiratory papillomatosis. Most HPV-related disease is vaccine-preventable.

In 2011, the United States was the first country to include routine vaccination for males in its national HPV vaccination program, including MSM through age 26 years.^3^ In late 2019, recommendations for catch-up HPV vaccination were harmonized to include people of all genders and sexual orientations through age 26 years.^5^

Although HPV infection is common among people of all genders in the United States, MSM and transgender women, particularly those who are living with HIV, bear a disproportionate burden of HPV infections and related diseases including anal cancers. Population-based data sources for examining health impacts of HPV vaccination among MSM are limited. Because nationally representative data are lacking, assessments often rely on special studies enrolling MSM or surveillance at sentinel sites such as sexually transmitted disease (STD) clinics. In addition, online surveys can identify and enroll large numbers of geographically dispersed lesbian, gay, bisexual, and transgender (LGBT) participants, although demographic characteristics of internet-based respondents tend to skew toward more participants of non-Hispanic white race/ethnicity, higher educational attainment, and higher household income levels than other types of study designs.^6–8^

In this paper, we review and summarize data relevant to HPV health outcomes and HPV vaccination among gay, bisexual, and other MSM and transgender women in the United States.

## Methods

We conducted a rapid review using the MEDLINE database to identify publications regarding HPV or HPV vaccination among gay, bisexual, or other MSM or transgender women, including information relevant to vaccination policy and the vaccination program in the United States. Search terms used were: ((HPV or human papillomavirus) and (vaccine or vaccines or vaccination)).mp. and (“men who have sex with men” or MSM or homosexual or gay or bisexual or sexual minorities or LGBTQ).ti,ab. Through June 7, 2021, we identified 379 publications of interest; we reviewed each abstract and selected 131 for more detailed review. We focused on six themes, including 1) burden of HPV infection and related diseases; 2) HPV vaccine efficacy among MSM; 3) HPV vaccination recommendations; 4) HPV vaccination coverage; 5) real-world HPV vaccine effectiveness and health impacts among MSM; and 6) HPV vaccination acceptability and interventions. After sorting by theme and including other relevant articles from the literature for context, we highlighted 72 publications in this narrative review.

## Results

### Burden of HPV infection and related diseases

MSM and transgender women bear a disproportionate burden of HPV infections, HPV-associated precancers, and HPV-associated cancers. Several meta-analyses of data from MSM have been conducted. A 2021 pooled analysis of data from 64 studies of both MSM and men who have sex with women, conducted in various countries, reported that anal prevalence of high-risk HPV types among men was 41.2% among 10,617 MSM versus 6.9% among 5,190 other men.^9^ A 2021 meta-analysis of data from 107 international studies comprising 36,773 MSM reported that pooled prevalence of anal, penile, oral and urethral HPV infections was 78.4% (95% confidence interval [CI]: 75.6%–81.0%), 36.2% (95% CI: 29.1%–44.0%), 17.3% (95% CI: 13.6%–21.7%) and 15.4% (95% CI: 7.8%–27.9%), respectively.^10^ In the United States, HPV seroprevalence was assessed in the National Health and Nutrition Examination Survey (NHANES); in this national survey, among 169 MSM who identified as gay/bisexual during 2003–2010, seroprevalence of quadrivalent HPV vaccine types 6/11/16/18 was 42.6%, higher than among other men (13.2%).^11^ For oral HPV infections, in a 2016 meta-analysis of data from five studies among HIV-negative MSM, pooled prevalence of high-risk HPV was 9.1% (95% CI: 4.0–14.2%).^12^ For histological high-grade AIN among HIV-negative men, a meta-analysis of six studies reported that pooled prevalence was 21.5% (95% CI: 13.7%–29.3%), with annual incidence of 3.3% and 6.0% in two estimates.^13^ MSM are about 20 times more likely than other men to develop anal cancer.^13^

MSM who are living with HIV bear a particularly high burden of HPV and related diseases. In a 2018 meta-analysis of data from 25 longitudinal studies, HPV incidence was approximately double and HPV clearance was approximately half among people with HIV compared to those without.^14^ In a 2021 pooled analysis of data on anal HPV from 64 international studies, prevalence of high-risk HPV types was 74.3% among 13,411 MSM living with HIV and 26.9% among 682 other men living with HIV.^9^ For oral HPV infections, in a 2016 meta-analysis of data from seven studies among MSM living with HIV, pooled prevalence of high-risk HPV was 16.5% (95% CI: 8.2–24.8%).^12^ For histological high-grade AIN, a meta-analysis of data from 17 studies reported that pooled prevalence was 29% among men living with HIV, with annual estimated incidence of 8.5% and 15.4% in two estimates.^13^ Incidence of anal cancer was significantly higher among MSM living with HIV than among HIV-negative MSM; a meta-analysis of 9 studies reported a pooled anal cancer incidence of 45.9 per 100,000 MSM living with HIV and 5.1 per 100,000 HIV-negative MSM.^13^

Data are limited regarding HPV-related health outcomes among transgender women in the United States. Many reports do not disaggregate results from MSM and transgender women, but some have reported an even higher burden of HPV infections among transgender women than among MSM. One study of unvaccinated young adults aged 18–26 years reported that prevalence of any HPV type in self-collected anal swabs was 88.6% among 44 transgender women, significantly higher than 70.9% among 855 MSM.^15^ A study of persons living with HIV found that among 22 transgender women, 95% had abnormal anal cytology results and 59% had a high-grade squamous intraepithelial lesion (HSIL) or squamous cell carcinoma (SCC); among 1448 MSM participating in the same study, 83% had abnormal anal cytology results while 39% had HSIL or SCC.^16^ Cancer registries do not typically collect information on sexual behavior or gender identity, and prevalence of anal cancer among transgender women is unknown.

### HPV vaccine efficacy among MSM

In the United States, two HPV vaccines have been licensed for use in males: quadrivalent HPV vaccine (4vHPV; Gardasil; Merck & Co.) and 9-valent HPV vaccine (9vHPV; Gardasil 9; Merck & Co.); only 9-valent vaccine is currently distributed.^3,17^ (A bivalent vaccine, Cervarix, manufactured by GSK, was licensed for use in females only.)^3^ In the pivotal randomized controlled trial of quadrivalent HPV vaccine in males aged 16–26 years, observed efficacy against external genital lesions associated with vaccine-type HPV was 90.4% (95% CI: 69.2%–98.1%) in the per-protocol population and 65.5% (95% CI: 45.8%–78.6%) in the intention-to-treat population.^18^ In a substudy of 602 MSM participating in this trial with 5 or fewer lifetime sex partners, efficacy against AIN associated with vaccine-type HPV was 77.5% (95% CI: 39.6%–93.3) in the per-protocol population and 50.3% (95% CI: 25.7%–67.2%) in the intention-to-treat population.^19^ The bridging immunogenicity study that was the basis for licensure of the 9-valent HPV vaccine included 313 MSM, >99% of whom seroconverted to all 9 HPV vaccine types following vaccination.^20^ Among MSM living with HIV aged 18–26 years, a single-arm clinical trial suggested that quadrivalent HPV vaccination could prevent HPV infection and high-grade AIN;^21^ however, among MSM living with HIV who had high rates of prior HPV infection, two randomized controlled trials did not find that vaccination prevented anal HPV infections or AIN in participants aged ≥27 years.^22,23^

### HPV vaccination recommendations in the United States

HPV vaccination is most effective when given before exposure to HPV through sexual activity, so ideally should be given to all persons in early adolescence, regardless of whether sexual orientation or gender identity have been established. Routine HPV vaccination is recommended at age 11 or 12 years; vaccination can be given starting at age 9 years.^3,5^

National recommendations by the Advisory Committee on Immunization Practices (ACIP) have changed over time based on new data and considerations since HPV vaccination was first recommended in 2006 for U.S. females.^3^ ACIP made HPV vaccination for males a permissive recommendation in 2009 and a routine recommendation in 2011.^3^ From 2011 to 2019, HPV vaccination was routinely recommended for both girls and boys at age 11 or 12 years, and, if they were not vaccinated previously, for females through age 26 years, males through age 21 years, and MSM and immunocompromised persons through age 26 years.^3^ Men who identify as gay or bisexual, or who intend to have sex with men, and transgender persons through age 26 years were specifically included beginning in 2016.^24^ In 2019, catch-up recommendations for HPV vaccination were harmonized through age 26 years for people of all genders.^5^ As of 2019, ACIP also recommends shared clinical decision-making^a^ between clinicians and patients regarding HPV vaccination for some adults aged 27–45 years.^5^ These recommendations from ACIP, adopted by CDC, are covered by most health insurance plans and incorporated into the national Sexually Transmitted Infections Treatment Guidelines in 2021.^25^

Health economic models have evaluated impact and cost-effectiveness of different vaccination strategies.^26^ Although HPV vaccination of girls is considered cost-effective, including males in national programs is relatively less cost-efficient than female-only vaccination in most models, particularly when males are protected indirectly through strong herd effects from female vaccination with high coverage.^26^ This is true even when all HPV-associated outcomes are included in models, although cost-effectiveness estimates are more favorable for both males and female vaccination when all outcomes (including oropharyngeal cancer, the most common HPV-associated cancer in males) are included. Most models have not included MSM specifically. Even in the context of a robust HPV vaccination program for girls, MSM would be less likely to benefit from herd immunity from a female vaccination program.^27^ One model showed that prioritizing vaccination for young MSM in Australia, either with or without a vaccination program for all males aged 15–26 years, would result in significant health benefits through prevention of anal cancers and anogenital warts.^28^ According to a systematic review of 17 studies and 12 underlying mathematical models of HPV vaccination for young adults, prioritized HPV vaccination for MSM aged 16–26 years may be a cost-effective option, albeit one with some implementation challenges, since early identification of this specific population can be difficult.^29^ A targeted literature review reported the incremental cost–effectiveness ratio (ICER) of HPV vaccination programs for MSM was $15,000–$43,000/quality-adjusted life-year [QALY] gained, and would be more cost-effective among younger MSM (aged <40 years) and those living with HIV.^30^

### HPV vaccination coverage

In the United States, where HPV vaccination is recommended for people of all genders through age 26 years, there is no targeted vaccination program for MSM, although some local jurisdictions have promoted HPV vaccination specifically for this population.^31^ In the National Immunization Survey of adolescents aged 13–17 years (NIS-Teen), overall, the percentage up-to-date for the series was 54.2% and ≥1-dose HPV vaccination coverage reached 71.5% in 2019.^32^ Coverage among males began to increase after 2011 when the routine recommendation was first made for males, and reached 69.8% for ≥1-dose and 51.8% up-to-date HPV vaccination coverage in 2019.^32^ In the National Health Interview Survey (NHIS) of U.S. adults, among males aged 19–26 years, ≥1-dose HPV vaccination coverage increased significantly from 2.1% in 2011 to 26.3% in 2018.^33^

Among MSM in the United States, there are no national estimates for HPV vaccination coverage. However, results from multi-site studies have suggested that coverage in these groups may be increasing over time. For example, data from National HIV Behavioral Surveillance, collected at three-year intervals among MSM in approximately 20 U.S. cities, have shown HPV vaccination coverage among MSM aged 18–26 years increasing from 5% in 2011 to 33% in 2017. ^34,35^^,36^ A 2020 meta-analysis of data on HPV vaccination among MSM reported an average HPV vaccination uptake of 38%.^37^ Of note, among 292 young adult MSM and transgender women with a documented history of ≥1 HPV vaccine dose, most (83%) were able to correctly self-report their HPV vaccination status in a clinical setting.^38^

Studies have been inadequate to estimate HPV vaccination coverage specifically among transgender women in the United States. In a survey conducted in 2014 of rural LGBT participants enrolled online that included 23 transgender women who were age-eligible for HPV vaccination, only one reported receiving a health-care provider recommendation for HPV vaccination, and only one reported ever receiving ≥1 dose of HPV vaccine.^39^ Such data suggest missed opportunities to address the need for HPV vaccination among transgender persons.

### Real-world HPV vaccine effectiveness and health impacts among MSM

Real-world HPV vaccine effectiveness (VE) for MSM has been demonstrated. In a study of 1767 MSM aged 18–26 years in three U.S. cities, quadrivalent vaccine-type combined anal and/or oral HPV prevalence was lower among vaccinated (22.9%) compared with unvaccinated (31.6%) participants; vaccine effectiveness of ≥1 HPV vaccine dose was 59% when first administered at age ≤18 years, but only 18% when the vaccination series was initiated at an older age.^40^ Among 687 participants in this study who also submitted penile specimens for HPV testing, VE against quadrivalent vaccine-type penile HPV was 85% among participants vaccinated at age ≤18 years.^41^ Vaccinated participants in this study had lower odds of quadrivalent HPV-type prevalence regardless of their reported sexual positioning practices (adjusted odds ratio 0.56; 95% CI: 0.34–0.92).^42^

In the United States and globally, there is compelling evidence of beneficial health impacts from HPV vaccination in females. A systematic review and meta-analysis of data from 65 studies on 60 million persons in 14 high-income countries showed that population-level impacts of HPV vaccination after 5–8 years of vaccination for girls and women included significant decreases in vaccine-type HPV prevalence, anogenital warts, and cervical precancers.^43^ Some countries have also included males in monitoring. Indirect vaccine impact among males overall through herd protection has been reported from HPV infection prevalence monitoring in NHANES in the United States and from genital wart surveillance in Australia; however, herd protection from female vaccination programs has provided limited benefit for MSM.^44–46^

Direct health impacts from vaccination of MSM also have been investigated in the United States and several other countries.^47^ At 27 U.S. STD clinics, prevalence of anogenital warts among all MSM decreased significantly during 2010–2016, from 6.2% to 2.9% (annual percent change −11.3%), with significant decreases across all age categories assessed.^48^ In British Colombia, Canada, age-adjusted rates of anogenital wart diagnoses decreased by 41% (adjusted relative risk 0.59, 95% CI: 0.38–0.91) in birth cohorts of MSM included in a clinic-based quadrivalent HPV vaccination program beginning in 2015.^49^ In England, an ecologic analysis of data from sexual health clinics revealed a slight decline in incidence of anogenital warts diagnoses among MSM aged 15–17 years in 2014–2017, from 129.9 to 112.2 per 100,000 population, respectively, after implementation of quadrivalent HPV vaccination program (a 13.6% decrease).^50^ In Australia, a repeated cross-sectional study of MSM aged 16–20 years before and after implementation of a school-based gender-neutral vaccination program showed a decrease in prevalence of quadrivalent vaccine-type penile HPV from 12% among 177 MSM in the pre-vaccination cohort to 6% among 179 MSM in the post-vaccination cohort.^51^

### HPV vaccination acceptability

Tailoring interventions to effectively prevent HPV-related disease among gay, bisexual, and other MSM and transgender women requires addressing facilitators and barriers to vaccination. The importance of a health-care provider recommendation has been reported consistently, and was the strongest predictor of vaccination in several studies of MSM aged 18–26 years.^52,53^ Other reported predictors have included younger age,^52^ having a healthcare visit in the past year,^35,36^ receiving other vaccinations recommended for MSM (i.e., hepatitis A and B),^35^ and living with HIV.^35,36,52^ A qualitative study of sexual minority men aged 18–26 years assessed information, motivation, and behavioral skills related to HPV vaccination, concluding that encouragement from a healthcare provider was the primary reason for receiving vaccination.^54^

Acceptability of HPV vaccination among gay, bisexual, and other MSM and transgender women is generally high. In an online survey, adult gay and bisexual men were significantly more interested in HPV vaccination than heterosexual men, and all were more willing to receive vaccination when framed as preventing cancer.^55^ In a systematic review and meta-analysis of 78 studies, mostly from the United States, average vaccine acceptability among MSM was 63% (median 72%, range: 30%–97%).^37^ In a different systematic review of 15 international studies of HPV vaccine acceptability, comprising 8658 MSM, pooled acceptability was 50% (95% CI: 0.27%–0.72%).^56^ Factors linked with greater acceptance included disclosure of sexual orientation to a health-care provider (odds ratio [OR] 2.38), vaccination against hepatitis A or B (OR 2.10), awareness of HPV (OR 1.85), having a college or higher degree (OR 1.62), knowledge of HPV (standard mean difference [SMD] 0.28), perceived susceptibility to HPV infection (SMD 0.31), and perceived severity of HPV-related disease (SMD 0.40); no significant relationship was identified with HIV status, and there was heterogeneity in results by gender identity.^56^

Because initial HPV vaccination recommendations differed for MSM and other men in the United States, catch-up vaccination for MSM aged 22–26 years initially relied on disclosure of same-sex sexual orientation or behavior to a vaccine provider. Results from an online survey showed that young men’s disclosure of male–male sexual behaviors to health-care providers predicted receipt of HPV vaccination.^57^ A study of 817 gay, bisexual, and other MSM aged 18–26 years who visited a health-care provider within the past year reported that 64.3% had disclosed, and 91.7% felt they could disclose if important to health.^58^ However, a 2018 study of Florida primary care physicians noted that only 13.6% routinely discussed both sexual orientation and HPV vaccination with male patients in this age range, and 24.5% discussed neither.^59^ In 2019, ACIP recommendations for HPV vaccination were harmonized across genders, obviating the need for disclosure for this purpose.^5^

Additional factors might contribute to health inequities experienced by sexual minorities. A study of HPV vaccination coverage among 1416 gay, bisexual, and other MSM and transgender women conducted in 2016–2018 in three U.S. cities noted significant differences in HPV vaccination coverage by city (range 33%–62%), age, race/ethnicity, and gender identity.^60^ Also, specific language or cultural differences may affect vaccination practices. For example, in a small study of 33 HIV-positive gay and bisexual men who were foreign-born Latino and interviewed in Spanish in 2015–2016, 15 (45%) had heard of HPV vaccine, and only one had received ≥1 dose.^61^

HPV vaccination may be offered in settings where MSM seek routine care, including HIV, STD, LBGTQ, primary care, or other clinics.^31,34,62,63^ A study assessing potential opportunities for HPV vaccination among gay, bisexual, and other MSM aged 18–26 years reported that 88.9% had accessed health care within the past year (i.e., had ≥1 visit to a health-care provider, HIV test, or syphilis test within the most recent 12 months);^63^ these results compared with observed HPV vaccination rates suggest that some opportunities for vaccination are being missed. Bundling HPV vaccination with HIV testing has been shown to be a feasible and acceptable way to increase coverage.^64^ Several other approaches to HPV vaccination interventions for young adult MSM have been attempted with some degree of success. In a qualitative study conducted in 2014–2015, young adult MSM recommended that, in addition to bundling vaccination with other health services and increasing awareness, HPV vaccination could also be facilitated by creative use of mobile technology.^65^ Accordingly, recent technological interventions have included mHealth, txt2protect, and Outsmart HPV. In a pilot study of the mHealth mobile health tool designed to facilitate HPV vaccination and education among young MSM in Boston, 23% of unvaccinated participants used the tool to obtain HPV vaccination.^66,67^ A pilot trial of txt2protect, a 9-month daily or monthly text-messaging-based program for young adult sexual minority men in Chicago, participant satisfaction was high and significantly more intervention participants (19%) initiated HPV vaccination compared with control participants (7%).^68^ In a pilot study of Outsmart HPV, a web-based intervention for U.S. gay and bisexual men enrolled via online advertisements, initiation of HPV vaccination was significantly higher among the intervention group (45%) than the control group (26%); a larger randomized controlled trial is underway.^69–71^ HPV vaccination interventions prioritizing transgender persons remain to be developed.

## Conclusions

In the United States, HPV vaccination is routinely recommended for all adolescents at age 11 or 12 years; also, catch-up vaccination through age 26 years is recommended for persons not previously vaccinated. Efforts are ongoing to increase HPV vaccination coverage among persons of all genders and to monitor impacts of HPV vaccination on health outcomes, including among populations disproportionately affected by HPV infection and HPV-related disease, such as gay, bisexual, and other MSM and transgender women. Bundling vaccination with other recommended health-care services for MSM and transgender women could be a feasible approach to increase vaccination in these populations since HPV vaccine acceptability is generally high. Increasing coverage of prophylactic vaccination among all persons before sexual exposure to HPV is cost-effective and expected to reduce the burden of HPV-related disease among all persons, regardless of gender identity, gender expression, or sexual orientation.

## Note

[a] In shared clinical decision-making, the decision about whether or not to vaccinate may be informed by the best available evidence on who may benefit from vaccination; the individual’s characteristics, values, and preferences; the health-care provider’s clinical discretion; and the characteristics of the vaccine being considered. HPV vaccination is not recommended for everyone aged 27–45 years.
